# Web-based survey among animal researchers on publication practices and incentives for increasing publication rates

**DOI:** 10.1371/journal.pone.0250362

**Published:** 2021-05-06

**Authors:** Susanne Deutsch, Miriam Heider, Silke Glage, André Bleich, René Tolba, Daniel Strech, Susanne Wieschowski

**Affiliations:** 1 Institute for Laboratory Animal Science & Experimental Surgery, RWTH Aachen University, Faculty of Medicine, Aachen, Germany; 2 Institute for Laboratory Animal Science, Hannover Medical School, Hannover, Germany; 3 Institute for Ethics, History, and Philosophy of Medicine, Hannover Medical School, Hannover, Germany; 4 QUEST Center for Transforming Biomedical Research, Berlin Institute of Health, Berlin, Germany; 5 Charité Universitätsmedizin Berlin, Berlin, Germany; University of Rennes 1, FRANCE

## Abstract

**Objectives:**

Publication bias, non-publication, and selective reporting of animal studies limit progress toward the 3Rs (Replacement, Reduction, and Refinement) that guide ethical animal testing, waste public resources, and result in redundant research, which collectively undermine the public’s trust in scientific reliability. In this study, we aimed to 1) validate findings from a previous follow-up study by our team that examined the publication rates of animal studies from protocol to publication and 2) identify incentives for improving publication rates in animal research.

**Methods:**

The researchers responsible for the animal proposals (n = 210) from our previous study were contacted as participants for a Web-based survey between October 2019 and April 2020. Question types varied between free text questions, answer options based on a 5-point Likert scale and closed yes/no questions.

**Results:**

In total, 78 researchers responsible for 101 of 210 animal study proposals participated, yielding a response rate of 48.1%. Results showed that the publication rate increased from 67% in our follow-up study to 70%. According to a 5-point Likert scale (from 1 = “not relevant” to 5 = “extremely relevant”), the most widely accepted suggestions for increasing publication rates were “Publication costs for open access journals are fully covered by funders or universities” (mean 4.02, SD 1.01), “Performance-based allocation of intramural funds for results reporting of animal research not supporting the initial hypothesis (including preprints and repositories)” (mean 3.37, SD 1.05), and “Researchers receive more information from scientific journals that also publish non-significant results” (mean 3.30, SD 1.02).

**Conclusion:**

While the extent of publication and publication practices have been thoroughly investigated for clinical trials, less data is available for animal research to date. Therefore, the study contributes in complementing the picture of publication practice in animal research. Suggestions from our survey may help improve the publication rates of animal studies.

## Introduction

Publication bias, non-publication, and selective reporting is a matter of concern to the scientific community [1–3]. Non-publication opposes the principle of the 3Rs (Replacement, Reduction, Refinement), which serves as an ethical guideline for using animals in experiments and informing experimental planning and procedures. Replacement refers to alternative methods that lead to the direct replacement of animals in experiments, Reduction aims at the minimization of the number of animals used in experiments and the term Refinement is used to describe experimental approaches that minimize pain, suffering and harm to experimental animals [4, 5]. Thus, non-publication may lead to the repetition of animal experiments by other researchers and thus a higher number of animals in experiments than necessary. Moreover, it wastes public funding because it leads to overlap in research endeavors and biases the literature toward desired outcomes (e.g., the efficacy of new drugs), ultimately hampering the public’s trust in scientific reliability [6–8].

While the extent of results publication has been thoroughly investigated for clinical trials, less data is available for the field of animal research [9]. In a 2012 survey targeting Dutch animal researchers, findings revealed that only 50% of all animal studies results were actually published [3]. A recently published study from our group tracking a representative sample of 210 animal studies at two German university medical centers from protocol to publication found a publication rate of 67%, employing a rigorous follow-up that included structured searches in multiple databases and a double-check for each individual follow-up by a second independent researcher [1]. We note, however, that we did not directly inquire about further publications (“researcher-check”), reasons for not publishing, or incentives to publish.

Our present study had two objectives. First, we aimed to identify the number of additional publications, as well as those publications that we had identified even though they did not actually match with the proposals through conducting a survey among the responsible researchers (“researcher-check”) for all 210 animal studies tracked from protocol to publication in the previous study. Second, within the same “researcher-check” we aimed to determine strategies for improving publication rates of animal studies.

## Material and methods

### Design

The present study used a closed Web-based survey designed in German, as all participants were German-speaking animal researchers. A web-based survey was utilized because it was considered the most effective and efficient way to reach animal researchers within two university medical centers simultaneously in a short period regardless of physical distance. As the two objectives of the survey were to validate the results of our previous follow-up study of animal studies [1] and to identify possible incentives for timely publication, the questionnaire was divided into two parts. The first part served to validate publication numbers and rates determined in the initial follow-up study, while the second part inquired about the practicality of various approaches for promoting timely results publication of animal studies. These approaches were identified in discussions with the project initiators of this study, who are experienced in publishing scientific surveys and interviews [2]. The researchers responsible for the animal proposals from the previous study were contacted as survey participants. Thus, the sample from the previous study was also used to assess factors that may help improve the publishing of animal research.

### Questionnaire

Only one survey question (or question block) was displayed per screen; resulting in a total of seven or eight questions distributed over a maximum of eight or nine screens (including the first page with survey instructions), respectively. The answer type varied between free text questions, answer options based on a 5-point Likert scale (from 1 = “not relevant” to 5 = “extremely relevant”) and closed yes/no questions. If the participant responded with “yes”, a free text field could be used to further specify the answer.

### Data collection

Data were collected between October 30, 2019 and April 28, 2020. Data collection for the previous study ended in June 2019, so there was a 3-month lag between the previous study and the current survey study. SoSci Survey software was used to design and conduct the survey (S1 Text). The data were processed and stored in pseudonymized form on a server at one of our study sites, as per the European Union’s 2016/679 regulations (General Data Protection Regulation). Only members of the study team had access to the data. All participants were informed about data storage and the lead investigators. Each participant decided to voluntarily participate in the survey by following the link to the survey provided and was informed that starting the survey will be regarded as consent to the collection, processing and storage of their data (implied consent). No incentives for participation were offered.

### Recruitment

All research proposals from our previous study were included (n = 210) [1], consisting of 105 proposals per study site. They had been stratified by year (end of approval, 2007–2013) and categorization of animals (rodents or non-rodents). For each year, 12 rodent and three non-rodent studies had been randomly selected, summing up to the total of 210 proposals. The principal investigator(s) and his or her deputy/deputies—as given on the proposals—were extracted for each proposal. This resulted in a range of 1–9 proposals per investigator (Table 1). Using this data, a contact list of all principal investigators and deputies was compiled. Then the email addresses were searched for through their affiliated institutions or online. If only addresses for the researchers’ general clinics or companies were available, the institutions were contacted for the researchers’ personal email addresses to ensure data protection. Once all email addresses were gathered, the researchers received an invitation email explaining the purpose of the survey and containing a personalized survey link.

**Table 1:** Frequencies of proposals per investigator.

Proposals per investigator	Frequency
1	140
2	49
3	16
4	9
5	7
6	2
7	1
8	2
9	1

At each study site, the survey was first pilot tested with 20 participants regarding wording and technical functionality. Reminders were sent one and three weeks after the initial invitation, respectively. The same procedure was followed with the remaining persons. If respondents named other persons to be contacted for more information on the proposals, invitations with new personalized survey links and two reminders were sent to these individuals as well. All responses to the survey, including those that were returned by email or telephone, were input into the data analyses. To conduct the survey, a functional email address with the domain of the respective study site was set up.

### Non-responder analysis

In April 2020, a non-responder analysis was conducted to obtain information on the reasons for non-participation. All participants were sent a brief email with the previously used functional email address asking why they did not participate and offering these options: a) lack of time to participate in the survey, b) proposals too old (documentation is no longer available), c) not responsible for the proposals and/or publications in question, and d) unsure about the legal consequences of participating in this survey. Selection of more than one option was possible, and a free text field was provided in attempt to gain additional insight. In the event that an individual still wanted to participate, a personalized survey link was also included.

### Statistical analysis

All descriptive data were analyzed using SPSS for Windows version 26 (IBM Corp., Armonk, NY).

### Registration and ethics statement

The study protocol was registered at the Open Science Framework (https://osf.io/u7erk/). This study was authorized by an Ethics Committee (8681_BO_K_2019) and the responsible data protection officer and information security officer were consulted. Reporting of results was based on the Checklist for Reporting Results of Internet E-Surveys (CHERRIES) [10].

## Results

### Response rate

After two reminders and a time lag of at least 20 weeks following the second reminder, a final response rate of 48.1% was reached. Specifically, for 101 out of 210 proposals we received responses from 78 participants regarding the two most important questions about publication status (i.e., whether there were any additional publications as well as whether we had identified publications that did not match the proposals). Response rates did not differ significantly between study site 1 (49/105 proposals; 46.7%) and study site 2 (52/105 proposals; 49.5%). Regarding the 172 proposals with confirmed conduction, the response rate was higher for proposals generating at least one results publication according to our previous study (54.4%, 62/114 proposals) than for those without any publications (48.3%, 28/58 proposals). Flow charts are provided in S1 and S2 Figs.

### Non-responder analysis

Of the initial 69 non-responders, 17 answered our follow-up email (25%). Four said they were not responsible for the proposals and/or publications in question (24%), four said the mentioned proposals were too old (24%), three had no time to complete the survey (18%), one was unsure about the legal consequences of participating in the survey (6%), one stated that participation was voluntarily (6%), two deferred participation to another person (12%), and three decided to participate in the survey after receiving the email (18%).

### Publication rates and numbers of publications

The first objective of our study, namely the "researcher check" for the total sample of 210 animal proposals from the previous study, sought to identify the number of additional publications, as well as those publications that we had identified even though they did not actually match with the proposals. The survey responses identified 172 proposals that were actually conducted, with a publication rate of 70%, slightly greater than the 158 proposals confirmed as conducted in the initial study with a publication rate of 67%. The average number of publications per conducted proposal was found to increase from 1.44 to 1.50. Of the 172 actually conducted proposals with at least one publication (120/172 proposals), 73% published at least one publication open access (88/120 proposals).

Of the 101 proposals with author responses to our survey, 85 led to no additional publications, 14 resulted in additional publications, and in two cases the publication found in the previous study was rejected, i.e. the results publications found in our literature search turned out not be matching the respective animal studies. This means, a confirmation rate of 84% of the results of our first follow-up.

For proposals that led to no publications according to our bibliographic search, 13.8% (8/58) resulted in at least one publication, as per responses to our survey. Of proposals with no results publications, survey responses provided reasons for 15 cases. The most frequent reasons were “methodological problems” (n = 6), followed by “study was not performed at all” (n = 6), “study was not finalized” (n = 2) and “patent issues” (n = 1). Raw data are provided in S1 Table.

### Approaches to increasing results publication of animal studies

**Fig 1:**
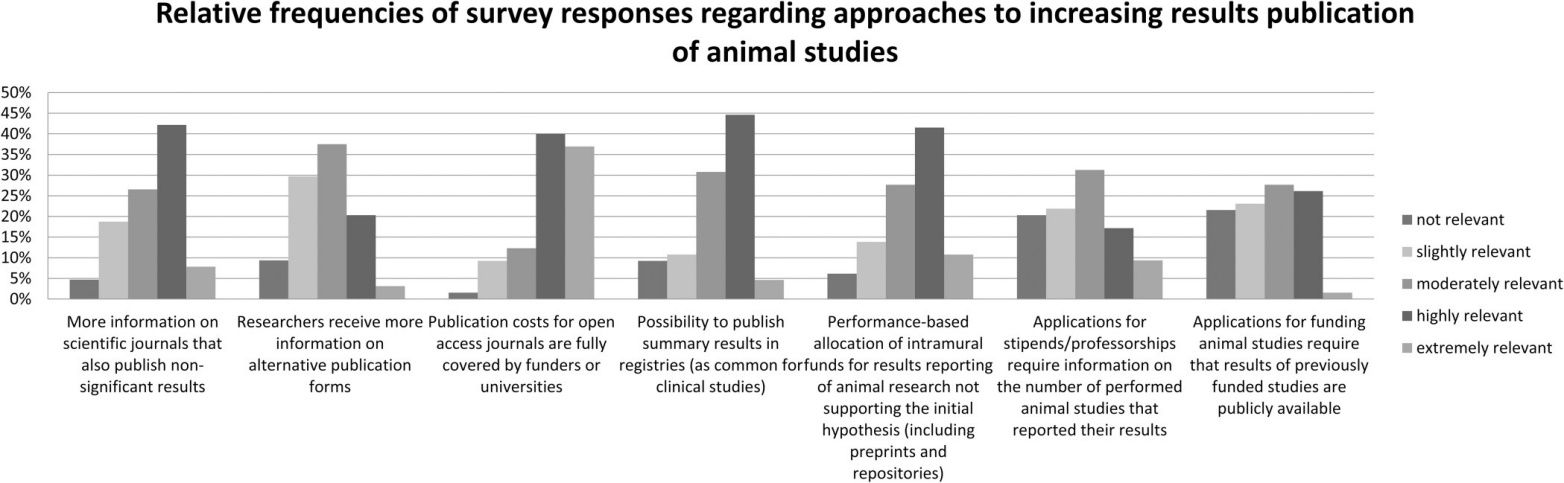
Relative frequencies of survey responses.

For the study’s second objective, researchers were asked about the relevance of various approaches for improving publication rates. Among the 78 respondents, 65 (83%) assessed the relevance of at least one such approach on a 5-point Likert scale of 1 to 5 (from 1 = “not relevant” to 5 = “extremely relevant”), of which “Publication costs for open access journals are fully covered by funders or universities” was rated as the most relevant (mean 4.02, SD 1.01), followed by “Performance-based allocation of intramural funds for results reporting of animal research not supporting the initial hypothesis (including preprints and repositories)” (mean 3.37, SD 1.05) and “Researchers receive more information on scientific journals that also publish non-significant results” (mean 3.30, SD 1.02). Ratings for the other options, such as “Possibility to publish summary results in registries (as common for clinical studies),” were only slightly above or below the indifference value of 3 (Table 2, Fig 1). In addition to the relevance of the approaches mentioned, the participants added the following aspects for improving publication rates in a free text field: "Results of animal experiments should always be published, even if only on the server of the performing faculty" (n = 1), "Scientific review of animal proposals and thus fewer and better experiments" (n = 1), "Publication of results must be available before approval of a further animal proposal" (n = 1) and "Allocation of funds partly only after publication (also in registers, etc.)" (n = 1). Raw data are provided in S2 Table.

**Table 2:** Ratings of approaches to increasing results publication of animal studies.

	**N**	**Percentiles**			**Mean**	**SD**
		25	50	75		
Researchers receive more information on scientific journals that also publish non-significant results	64	3.00	3.50	4.00	3.30	1.02
Researchers receive more information on alternative publication forms such as preprint servers (e.g., bioRxiv, without peer-review), F1000Research (post-publication review) or repositories (e.g., OSF)	64	2.00	3.00	3.00	2.78	0.98
Publication costs for open access journals are fully covered by funders or universities	65	4.00	4.00	5.00	4.02	1.01
Possibility to publish summary results in registries (as common for clinical studies)	65	3.00	3.00	4.00	3.25	1.03
Performance-based allocation of intramural funds (German: Leistungsorientierte Mittelvergabe/LOM) for results reporting of animal research not supporting the initial hypothesis (including preprints and repositories)	65	3.00	4.00	4.00	3.37	1.05
Applications for stipends/professorships require information on the number of performed animal studies that reported their results	64	2.00	3.00	4.00	2.73	1.24
Applications for funding animal studies require that results of previously funded studies are publicly available	65	2.00	3.00	4.00	2.63	1.14

## Discussion

This survey study had two objectives. The first was to validate the results of the previous study tracking animal research from protocol to publication [1] and the second was to identify possible incentives for timely results publication of animal studies. In total, 78 persons responsible (as indicated on the animal proposals from the previous study) participated in the survey, yielding a final response rate of 48.1% (i.e. for 101 proposals of the total sample of 210 proposals the minimal set of questions regarding publication status had been answered). Based on the survey, we identified additional proposals with confirmed conduction yielding published results, thereby increasing the total publication rate of the original sample from 67% to 70% and the mean number of publications per proposal from 1.44 to 1.50. These findings align with that from a recently published study tracking a selection of animal study protocols approved at the university medical center in Utrecht, The Netherlands, wherein the publication rate was 60% [11], supporting our contention that a substantial fraction of animal studies remain unpublished. Failure to publish results from animal studies is a matter of concern [1, 3, 6, 7, 12], necessitating concrete measures and incentives to improve publication practices. For this reason, we asked the researchers to rate the relevance of possible incentives for overcoming this problem. The relevance ratings fell within a narrow middle range (medians between 3 and 4 on a 5-point Likert scale of 1 = “not relevant” to 5 = “extremely relevant”). Therefore, a more in-depth interview study is required to take a closer look at this. However, participants were in broad agreement that compensation for publishing in open access journals, allocation of internal research funds for publishing results that stand at odds with the original or prevailing hypothesis, and more information on journals publishing non-significant results could help increase publication rates. To what extent the approaches for improving publication rates were actually involved in the publication rate observed for the 210 proposals studied, will be part of a subsequent study.

The European Commission states that “Nowadays, it is widely recognized that making research results more accessible contributes to better and more efficient science, and to innovation in the public and private sectors” [13]. Open access publishing is of particular importance for the dissemination of research results, and being compensated for the open access fee is desired not only by our participants but also by medical informatics specialists in a previous study [14]. Moreover, major funding organizations like the German Research Foundation (Deutsche Forschungsgemeinschaft) (https://bit.ly/32Qg27L) and the Federal Ministry of Education and Research (Bundesministerium für Bildung und Forschung, https://bit.ly/2ZWWSet) recognize this is needed by many researchers. Grant recipients of the aforementioned major funding organizations are also required to publish their project results open access for the purpose of science-appropriate communication. Thus, publications in subscription-based journals are no longer what is required and expected. This hypothesis is also strengthened by our data, which show that almost three quarters of the actually conducted proposals with at least one publication have published at least one publication open access. Furthermore, multidisciplinary journals that ask peer-reviewers to not review the relevance of results require open-access fees. In addition to that, results from completed projects that currently lay dormant, because researchers lack sufficient funding, may be published through post-grant funds for open access publishing (https://bit.ly/2ZWotMO). Thus, open access publishing is an important instrument for strengthening scientific discourse, promoting scientific progress, and making scientific findings available to the scientific community, as well as to the general public.

The value of non-significant results is now acknowledged by many in the scientific community, as such information can prevent unnecessary animal experiments from being conducted, offer suggestions for methodological improvements, and even lead to alternative hypotheses. Indeed, accepting non-significant results that do not confirm the original hypothesis should be encouraged, and studies that are well planned and conducted should contribute to scientific knowledge regardless of the outcome. One way to promote this is to submit manuscripts without a results section so that editors and reviewers judge the research simply by its background and methodology [3]. Considering many journals currently publish studies despite their findings, dissemination of non-significant results should not remain a challenge. Nevertheless, this practice does not appear to be widely known, on account that our participants wanted more information on journals that publish non-significant results. In addition to information about ways to publish non-significant results, further incentives should be established to encourage publication of all results. For example, performance-based allocation of intramural funds (in German: Leistungsorientierte Mittelvergabe) for publishing results that do not support the study’s hypothesis was seen as a favorable option, according to our survey. In contrast, participants seemed less enthused about the requirements for information on how many animal studies have actually been published to apply for scholarship or professorship, and that results from previously funded studies be made available to the public to apply for funding. For the latter, it is possible that this is because of the fact that acceptance and publishing of manuscripts can be delayed by lengthy peer reviews in standard journals.

At the moment, it is still difficult to publish non-significant results from animal experiments in high-impact journals; therefore, it is likely that all researchers will encounter this issue at some point in their careers. Generally speaking, funding and professorships are awarded to researchers who publish in high-impact journals. For this reason as well as the often prolonged publication process, publishing findings from previous studies to apply for funding applications are not always realistic. The recently published “Hong Kong Principles for Assessing Researchers” highlights the need for change and recommends that researchers be evaluated on trustworthiness, rigor, and transparency [15].

In addition to standard peer-reviewed journals, there are many new outlets for disseminating research findings, such as preprint servers (e.g., bioRxiv) or repositories such as Open Science Framework (OSF) and FigShare [16]. These create new possibilities for partial circumvention around obstacles involved in publishing. However, to ensure that peer-reviewed journals do not remain the dominant publication medium revered by the scientific community, these alternative outlets must be encouraged. According to our survey, these outlets are not widely known, nor is their potential appreciated. This could also be remedied by the possibility of publishing study results in animal study registries, a common practice in clinical research [2, 17, 18].

The present study identified various incentives for improving the publication rates of animal studies. To provide a more effective pipeline for disseminating all types of findings in animal research, future studies should extend the survey sample to include other institutions and stakeholders, since the participants of this study were members of only two German university medical centers. Nevertheless, the study contributes in complementing the picture of publication practice in animal research and the findings identify possible approaches and their relevance to improve publication practices in animal research.

## Supporting information

S1 Text Final survey. (DOCX)

S1 Fig Flow chart 1. Total sample of animal proposals (n = 210 proposals).
(TIF)

S2 Fig Flow chart 2. Actually conducted animal proposals after researcher check (n = 172 proposals).
(TIF)

S1 Table Raw data 1. Publication rates and numbers of publications (data that may identify individual participants are redacted).
(XLSX)

S2 Table Raw data 2. Approaches for timely publication of results from animal studies (data that may identify individual participants are redacted).
(XLSX)
