# Supplementary material for: Web-based survey among animal researchers on publication practices and incentives for increasing publication rates
Source: PLoS One. 2021 May 6;16(5):e0250362. doi: 10.1371/journal.pone.0250362 (PMC8101964; doi:10.1371/journal.pone.0250362)
Supplement: S1 Text — (DOCX) [file pone.0250362.s001.docx]

# Protocol EMBARC survey/author check

## Procedure

We included all proposals from our previous follow-up study (1), i.e. 105 proposals per study site that were stratified by year (end of approval, 2007–2013) and categorization of animals (rodents/non-rodents). For each year 15 proposals including 12 rodent and 3 non-rodent studies were in this sample.

For each proposal, we extracted the principal investigator(s) and his or her deputy/deputies as given on the proposals. Using this data, we created a contact list containing each investigator assigned to all proposals he/she was named on. We searched for e-mail addresses of those persons, first via the institution that the proposal was originated from and, if persons were no longer affiliated with that institution, we used a web search to find them. If no personal address was available, but only general clinic or company addresses, we first contacted this address and asked for personal email addresses for our survey to protect the participants’ personal data.

At each study site, we performed a pilot survey with 20 contact persons from each sample to exclude technical and understanding problems in the survey. Reminders were sent one and 3 weeks after the initial invitation, respectively. If respondents asked back via e-mail and answered survey questions via phone or e-mail, we also included their answers in our sample. If respondents named other persons to be contacted for more information on the proposals we had named in our e-mail, we sent invitations with new personalized survey links to those other persons including two reminders, if necessary.

SoSci Survey software was used to design and conduct the survey. The data was stored and processed in pseudonymized form on the server of one of the study sites in accordance with the data protection regulations. Only direct members of the study team had access to the pseudonymized data.

## Survey-Items

| Sind Aktenzeichen genannt, für deren Versuchsvorhaben Sie nicht (stellvertretend) verantwortlich sind?  - Ja 🡪 bitte Aktenzeichen angeben  - Nein |
| --- |

This question was only asked at one study site:

| Sind Aktenzeichen genannt, deren Versuchsvorhaben nicht durchgeführt worden sind (d.h. es wurden keine Tiere verbraucht)?  - Ja 🡪 bitte Aktenzeichen angeben  - Nein |
| --- |

| Im E-Mail-Anschreiben haben wir Ihnen die Ergebnispublikationen genannt, die wir für Ihren Tierversuchsantrag gefunden haben. Gibt es WEITERE Ergebnispublikationen zu dem betreffenden Antrag  - Ja 🡪 bitte weitere angeben (Aktenzeichen des Antrags + Link oder DOI)  - Nein |
| --- |

| Haben wir NICHT ZUTREFFENDE Ergebnispublikationen identifiziert, d.h. Publikationen, die nicht die Ergebnisse des betreffenden Antrags berichten?  - Ja 🡪 bitte angeben, welche Publikationen nicht passen (Link oder DOI)  - Nein |
| --- |

| Haben Sie Methoden oder Ergebnisse ihres Tierversuchsantrages über andere Wege/Medien veröffentlicht, wie z.B. Preprints, Repositorien (Open Science Framework), Register etc.?  - Ja 🡪 bitte Aktenzeichen des Antrags + Link oder DOI angeben  - Nein |
| --- |

| Folgende Frage betrifft nur Anträge, für die wir keine Publikation gefunden haben (siehe Einladungs-Email).  Wir konnten über Literaturrecherchen KEINE Ergebnispublikationen zu einem oder mehreren Anträgen finden. Gibt es DENNOCH Ergebnispublikationen zu den betreffenden Anträgen?  - Ja 🡪 bitte angeben (Aktenzeichen des Antrags + Link/DOI)  - Nein  - Nicht zutreffend |
| --- |

| Folgende Frage betrifft nur Anträge, für die wir keine Publikation gefunden haben (siehe Einladungs-Email).  Welche GRÜNDE waren ausschlaggebend für die Nichtpublikation von Ergebnissen?  - Freitext: |
| --- |

| Wie bewerten sie die praktische **Relevanz** der folgenden **Ansätze** zur Förderung zeitnaher Ergebnispublikationen tierexperimenteller Studien? (Score: 1-5 von gar keine Relevanz bis sehr hohe Relevanz)   - Mehr Informationen zu Fachzeitschriften, die auch nicht-signifikante Ergebnisse aus Tierexperimenten berichten (z.B. PLoS One) - Mehr Informationen zu anderen Publikationsformaten wie Preprint Server (z.B. bioRxiv, ohne peer-review), F1000Research (post-publication review), oder Repositorien (z.B. OSF). - Übernahme der Publikationskosten für Open Access Journale durch die Hochschulen oder Drittmittelgeber - Möglichkeit der Veröffentlichung von kurzen Zusammenfassungen der Ergebnisse („Summary Results“) in Registern für Tierforschung (ähnlich wie bei klinischen Studien) - Spezielle Leistungsorientierte Mittel (LOM) für die Veröffentlichung von tierexperimentellen Ergebnissen, die die ursprüngliche Hypothese nicht unterstützen (inkl. Publikationen auf Preprint-Servern oder Repositorien etc.) - Bewerbungsverfahren um Stipendien/Professuren erfordern die Angabe der Anzahl genehmigter Tierversuchsanträge mit/ohne Ergebnispublikation - Antragsverfahren zur Förderung von Tierstudien durch z.B. DFG/BMBF sind nur möglich, wenn Ergebnisse ehemals geförderter Tierstudien veröffentlicht wurden - Weitere Ansätze: (Freitext) |
| --- |

English translation for this manuscript

| Are reference numbers mentioned for which you are not (deputy) responsible?  - Yes 🡪 please name reference number(s)  - No |
| --- |

This question was only asked at one study site:

| Are reference numbers mentioned for studies that were not performed (i.e. no animals were used)?  - Yes 🡪 please name reference number(s)  - No |
| --- |

| In our invitation email we named those results publications that we could identify for your proposal(s). Are there FURTHER results publications for this proposal/these proposals?  - Yes 🡪 please name further (reference number(s) + link or DOI)  - No |
| --- |

| Did we name NON-MATCHING results publications, i.e. publications that do not report results of the respective proposal?  - Yes 🡪 please name non-matching publications (link or DOI)  - No |
| --- |

| Have you published methods or results of your proposal through other means/media?  - Yes 🡪 please name further reference number(s) + link or DOI  - No |
| --- |

| The following question only applies to proposals for which we could not identify any results publication (see invitation email).  Via literature search we were not able to find results publication for one or more of your proposals. Are there results publications that we have overlooked?  - Yes 🡪 please name (reference number(s) + link or DOI)  - No  - Not applicable |
| --- |

| The following question only applies to proposals for which we could not identify any results publication (see invitation email).  What REASONS were decisive for the non-publication of results?  - free text: |
| --- |

| How do you rate the practical relevance of the following approaches to increase timely results publication of animal studies? (Score: 1-5 from not relevant to extremely relevant)   - Researchers receive more information on scientific journals that also publish non-significant results (e,g, Plos One) - Researchers receive more information on alternative publication forms such as preprint servers (e.g., bioRxiv, without peer-review), F1000Research (post-publication review) or repositories (e.g., OSF) - Publication costs for open access journals are fully covered by funders or universities - Possibility to publish summary results in registries (as common for clinical studies) - Performance-based allocation of intramural funds (German: Leistungsorientierte Mittelvergabe/LOM) for results reporting of animal research not supporting the initial hypothesis (including preprints and repositories) - Applications for stipends/professorships require information on the number of performed animal studies that reported their results - Applications for funding animal studies require that results of previously funded studies are publicly available - Further approaches: (free text) |
| --- |

1. Wieschowski S, Biernot S, Deutsch S, Glage S, Bleich A, Tolba R, et al. Publication rates in animal research. Extent and characteristics of published and non-published animal studies followed up at two German university medical centres. PLoS One. 2019;14(11):e0223758.
