# Supplementary figures and images for: Web-based survey among animal researchers on publication practices and incentives for increasing publication rates
Source: PLoS One. 2021 May 6;16(5):e0250362. doi: 10.1371/journal.pone.0250362 (PMC8101964; doi:10.1371/journal.pone.0250362)

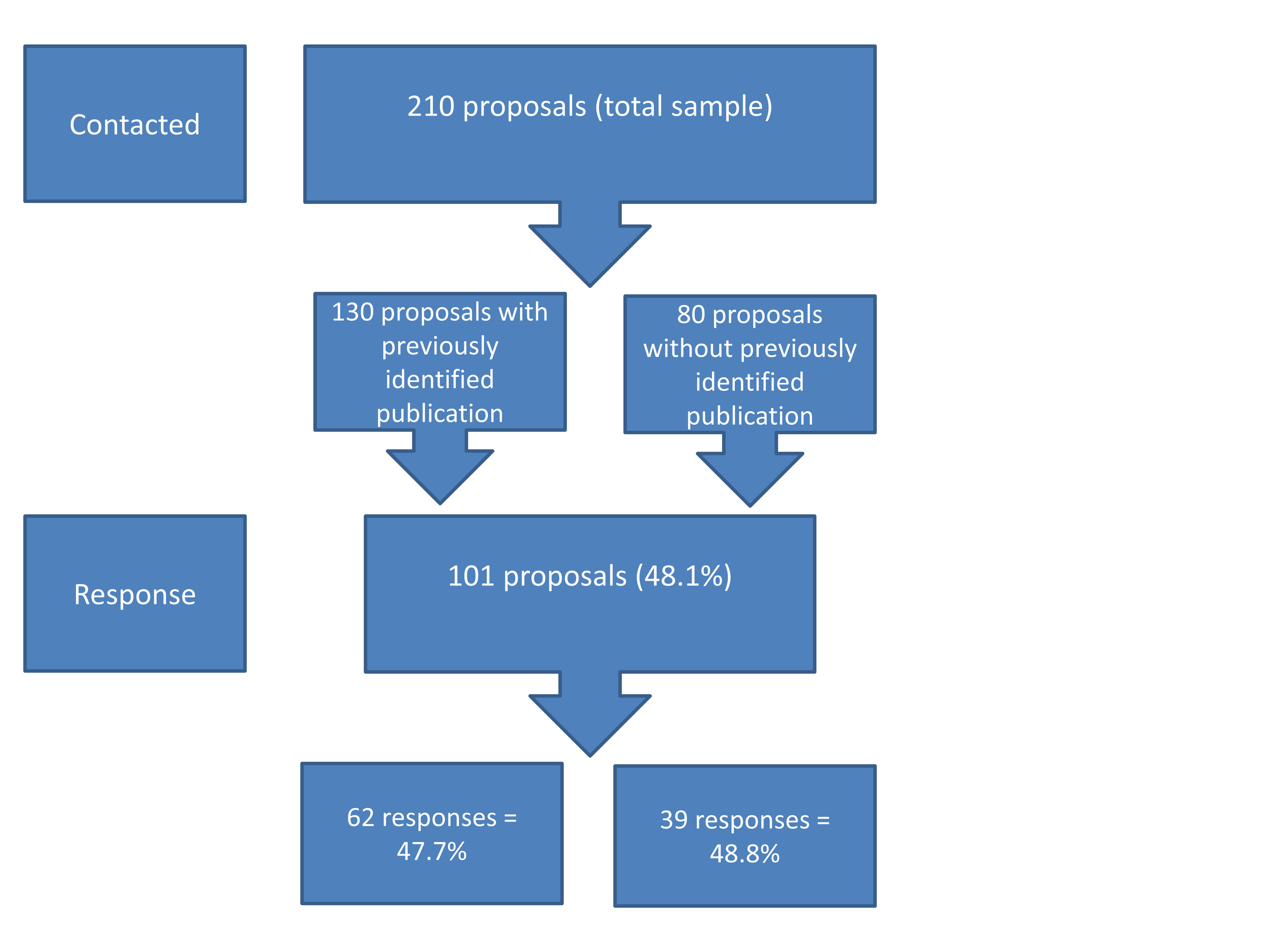

Supplement: S1 Fig — Total sample of animal proposals (n = 210 proposals). (TIF) [file pone.0250362.s002.tif]

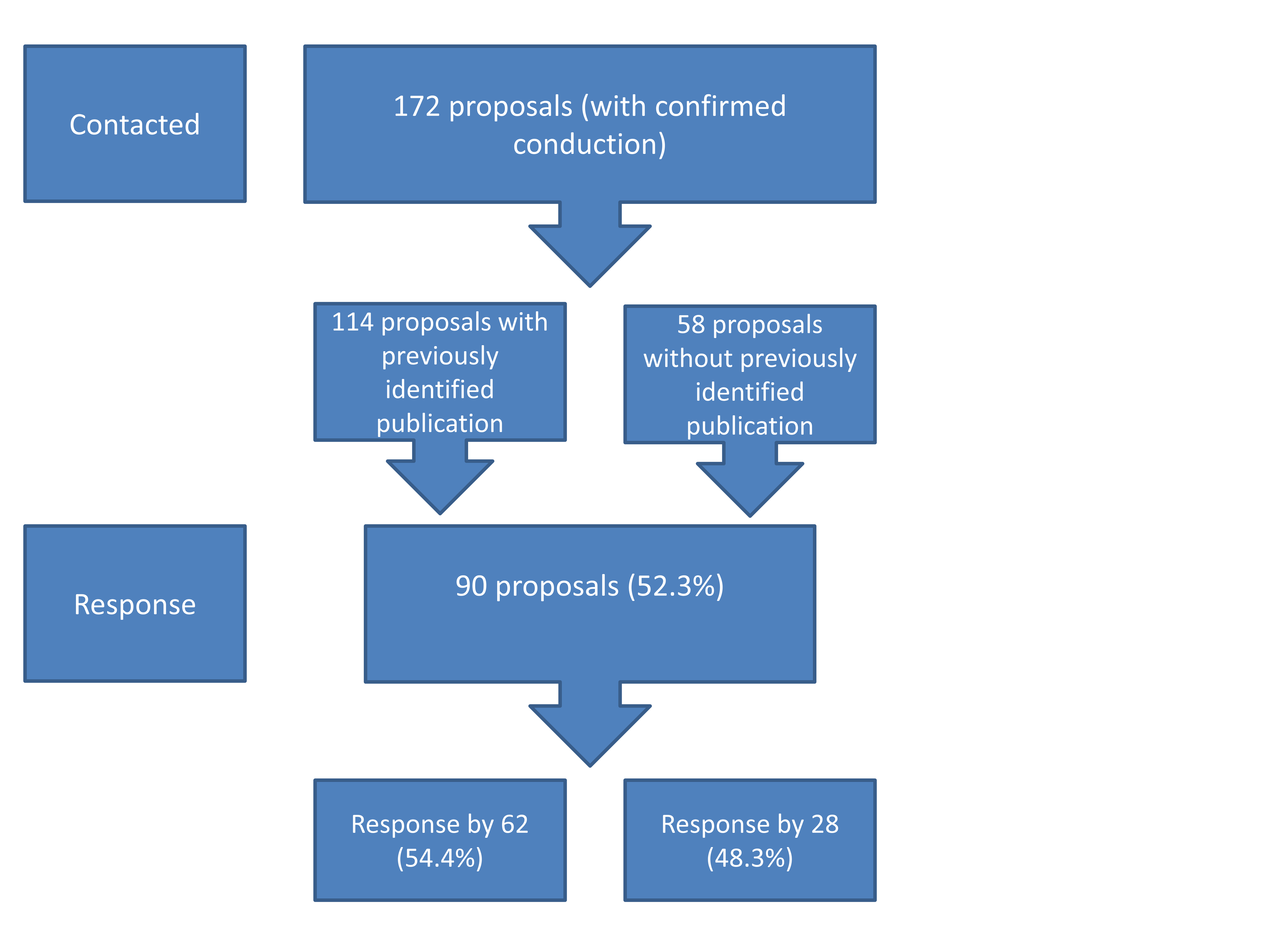

Supplement: S2 Fig — Actually conducted animal proposals after researcher check (n = 172 proposals). (TIF) [file pone.0250362.s003.tif]
